# Analysis of ADP-glucose pyrophosphorylase expression during turion formation induced by abscisic acid in *Spirodela polyrhiza *(greater duckweed)

**DOI:** 10.1186/1471-2229-12-5

**Published:** 2012-01-11

**Authors:** Wenqin Wang, Joachim Messing

**Affiliations:** 1 Waksman Institute of Microbiology, Rutgers University, 190 Frelinghuysen Road, Piscataway, NJ 08854, USA; 2 Department of Plant Biology and Pathology, Rutgers University, 59 Dudley Road, New Brunswick, NJ 08901, USA

**Keywords:** Duckweed, *Spirodela*, Starch, Turion, ADP-glucose pyrophosphorylase

## Abstract

**Background:**

Aquatic plants differ in their development from terrestrial plants in their morphology and physiology, but little is known about the molecular basis of the major phases of their life cycle. Interestingly, in place of seeds of terrestrial plants their dormant phase is represented by turions, which circumvents sexual reproduction. However, like seeds turions provide energy storage for starting the next growing season.

**Results:**

To begin a characterization of the transition from the growth to the dormant phase we used abscisic acid (ABA), a plant hormone, to induce controlled turion formation in *Spirodela polyrhiza *and investigated their differentiation from fronds, representing their growth phase, into turions with respect to morphological, ultra-structural characteristics, and starch content. Turions were rich in anthocyanin pigmentation and had a density that submerged them to the bottom of liquid medium. Transmission electron microscopy (TEM) of turions showed in comparison to fronds shrunken vacuoles, smaller intercellular space, and abundant starch granules surrounded by thylakoid membranes. Turions accumulated more than 60% starch in dry mass after two weeks of ABA treatment. To further understand the mechanism of the developmental switch from fronds to turions, we cloned and sequenced the genes of three large-subunit ADP-glucose pyrophosphorylases (*APLs*). All three putative protein and exon sequences were conserved, but the corresponding genomic sequences were extremely variable mainly due to the invasion of miniature inverted-repeat transposable elements (MITEs) into introns. A molecular three-dimensional model of the SpAPLs was consistent with their regulatory mechanism in the interaction with the substrate (ATP) and allosteric activator (3-PGA) to permit conformational changes of its structure. Gene expression analysis revealed that each gene was associated with distinct temporal expression during turion formation. *APL*2 and *APL*3 were highly expressed in earlier stages of turion development, while *APL*1 expression was reduced throughout turion development.

**Conclusions:**

These results suggest that the differential expression of *APL*s could be used to enhance energy flow from photosynthesis to storage of carbon in aquatic plants, making duckweeds a useful alternative biofuel feedstock.

## Background

Duckweed is an aquatic plant seen on water surfaces in many locations in the world. Because it consists mainly of a leaf-like body that performs photosynthesis, it is probably the most efficient multicellular biological solar energy converter that we have. Its structure during this stage of the life cycle is referred to as fronds. Greater duckweed or *Spirodela polyrhiza *is extremely simple with only one frond (merging leaf and stem) and some roots into a compact structure. Fronds grow vegetatively and can increase biomass rapidly, lowering carbon dioxide in the air and reducing nitrogen and phosphor in the water [1]. Many species of duckweeds can double their biomass every 2 or 3 days [2,3]. In addition, the tiny and free-floating duckweeds need very little amount of lignin to support their growth [4]. On the contrary, they might save the extra energy to synthesize more protein and carbohydrate. *Spirodela polyrhiza *has low amount of lignin [4], which contains 29.1% of protein [2] and up to 70% carbohydrate in dry weight [5]. The relatively easy harvesting process compared to algae is to skim of the floating fronds by net or collect them at the outlet of water by a grid [5].

There are conditions like temperature shifts due to seasons that can cause a morphological change to a different structure, called turions. Many species of the subfamily *Lemnoideae *can produce this kind of dormant fronds, which are characterized by more starch, smaller vacuoles and air space [6,7]. This developmental change is also accompanied by a shift in metabolism. The energy harvested during photosynthesis is shifted to starch biosynthesis, resulting in the accumulation of starch in turions. Because the volume of intercellular air space shrinks and starch increases the density of the tissue, it can sink to the bottom of waters where the organism can survive even if the top of the water freezes. Turions can change back to fronds vegetatively using the starch as an energy source, demonstrating a highly evolved adaptation to the environment. Because fronds have little lignin, which would interfer with the digestion of the carbohydrate fraction of biomass, and turions have high starch content, duckweed might also be suitable as an alternative source of bioenergy. Whereas cellulose is a crystalline, compact and structural compound resistant to biological attack and enzymatic degradation, starch is readily digested. Even though many advances over the past years have been made in the commercialization of cellulosic biomass [8], the cost of producing equal amounts of ethanol from cellulosic biomass is still much higher than production directly from starch [9]. Therefore, growing attention is being devoted to use duckweeds as a source of carbon compounds and convert duckweed biomass into bio-ethanol [10]. Fronds growing in swine wastewater contain 45.8% (dry weight) of starch. Moreover, 50.9% of the original dry biomass can be enzymatically hydrolyzed into a reducing sugar, which contributes to 25.8% fermented ethanol of dry biomass [10].

Recent studies have focused on the influence of various environmental conditions for turion formation or germination [11-15], the sensitivity threshold of ABA for turion formation [13,16] and the different structure (air space, vacuole, starch and cell wall) of fronds and turions [7]. On the other hand, information of starch content, granule size, and derivation of starch granules involvement with turion formation, which is critical to explore the potential biofuel of duckweed, is less well understood.

The pathway of starch synthesis is very complex, but ADP-glucose pyrophosphorylase (AGPase) plays a pivotal role in regulating starch levels and in determining patterns of starch deposition in plants. This enzyme comprises two identical large subunits (APLs) and two same small subunits (APSs) in angiosperms, each of which is encoded by distinct genes. Even though the roles of each AGPase subunit in the enzyme are not clear, it is generally proposed that APLs modify the response to allosteric regulators, whereas APSs act as the catalytic part [17]. Recent studies suggest that AGPase are usually in plastidial forms except for a cytosolic one in cereal endosperms [18,19]. Here, we compared the distinctive attributes between fronds and turions in *S. polyrhiza *and investigated starch production during development upon induction with abscisic acid (ABA), a plant hormone. To gain further insight into the function of the large subunit of AGPase (*APLs*) in starch synthesis as well, we cloned the *Spirodela *genes, analyzed them, and quantified their expression, which will allow in the future targeting expression of transgenes.

## Results

### Turion induction with ABA

**Figure 1:**
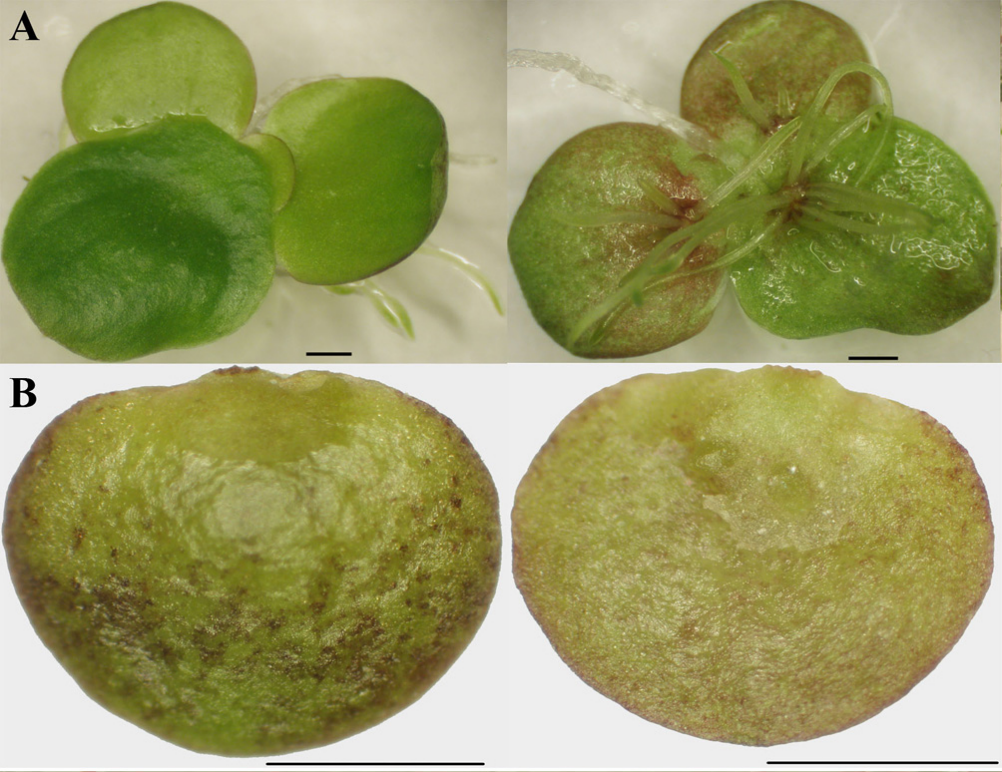
**Morphological comparison of frond and turion formed after 14 days of ABA treatment**. **a**) dorsal and ventral frond; **b**) dorsal and ventral turion. Bars = 1 mm.

**Figure 2:**
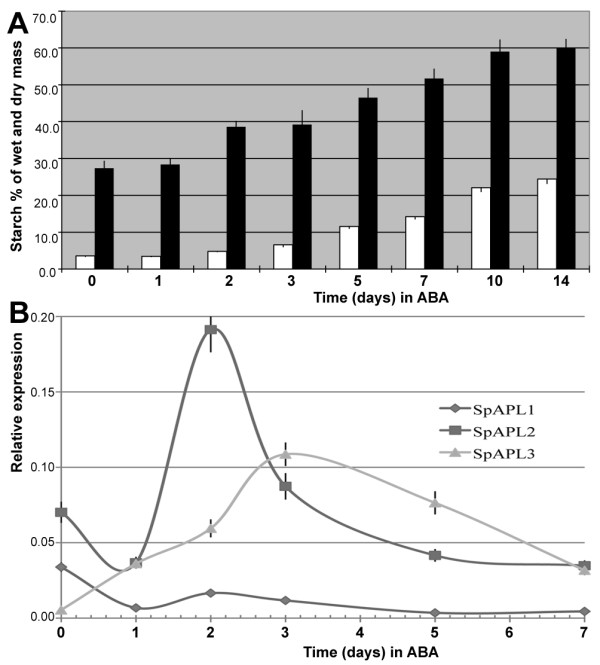
**Starch accumulation and expression of the APL genes during turion development**. **a**) White bars stands for wet tissue and black bars for dry tissue. Y-axis shows starch content (mg) for every 100 mg wet tissue or dry tissue. **b**) qPCR was used to quantify expression of APLs based on RNA from 0, 1, 2, 3, 5, 7 days of ABA treatment. Standard error was shown by vertical bar. Sample collections for starch analysis and *APLs *expression quantification were listed in Table 1.

*Spirodela polyrhiza *was grown under controlled light conditions as described under Methods. Fronds were harvested and examined under a dissecting microscope. Dividing fronds, representing single leaf-like bodies, were connected, thin, and elliptical (~8 mm in length and ~6 mm in width). The top of fronds was bright green, whereas the bottom extended a few roots that were submerged into water (Figure 1a). Continued growth in the presence of ABA gave rise to turion formation with different morphological features (Figure 1b). After 5 days of ABA application a significant shift to starch accumulation took place in samples collected from both wet and dry tissues. Starch accumulation during turion development exhibited a characteristic pattern. There was a progressive increase of starch from 5 days to 10 days after ABA application and after 14 days, the starch content became almost stable. The final starch content in turions for wet tissues was 24.4%, which corresponds to 60.1% in dry mass (Figure 2a). Turions were also harvested and examined under a dissecting microscope. They appeared thicker and smaller in nearly round shape (~2 mm in length and ~3 mm in width). Turions were dark green, spotted with many anthocyanin pigments, and retained only rudimentary roots that are not visible by naked eye (Figure 1b).

**Figure 3:**
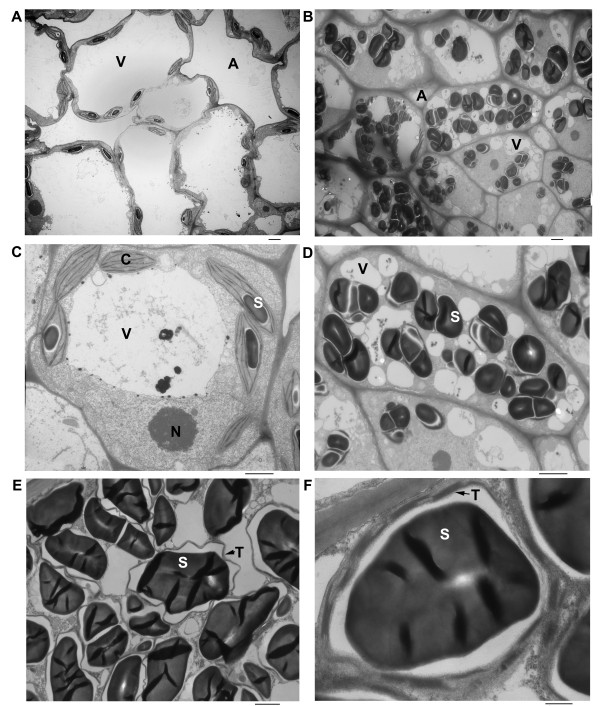
**Microscopic study**. **a**) Transmission electron microscopic (TEM) picture of frond cells with lower magnification, Bars = 2 μm; **b**) TEM picture of turion cells with lower magnification, Bar = 2 μm; **c**) TEM picture of a frond cell with higher magnification, Bar = 2 μm; **d**) TEM picture of a turion cell with higher magnification, Bar = 2 μm; **e**) TEM picture of a section of a turion cell with higher magnification, Bar = 2 μm; **f**) TEM picture of a section of a turion cell with the highest magnification, Bar = 500 nm. Abbreviations are chloroplast (C), starch granule (S), vacuole (V), intercellular air space (A), thylakoid membrane (T), nucleus (N).

**Figure 4:**
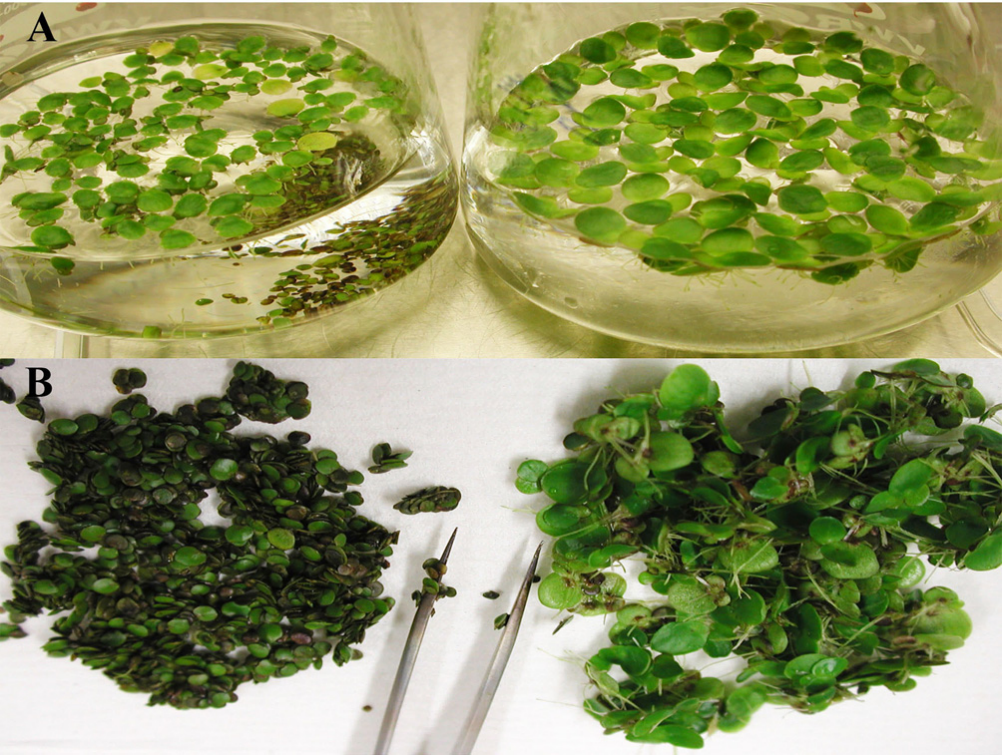
**Turion formation induced by ABA**. **a**) Turions (left panel) on the bottom and fronds swimming with roots down (right panel) in flasks; **b**) turions (left) and fronds (right) placed on filter paper. Bars = 1 mm.

Frond samples were then examined by electron microcopy. The frond cell had normal discal chloroplasts with a few small starch grains (Figure 3a and 3c). Most frond cells contained a single larger vacuole and bigger intercellular air space, while turion cells have multiple smaller vacuoles and bigger air space between cells (Figure 3b). The turion cell accumulated many starch granules, which almost occupied 1/4-2/3 of cell volume (Figure 3b and 3d). The kidney-shaped starch granule was surrounded with stacks of thylakoid membranes in chloroplasts (Figure 3e and 3f). The increased starch granules at the expense of the vacuolar expansion also contributed to the distortion of chloroplasts (Figure 3e) and a shift in tissue density that caused turions to sink to the bottom of liquid medium (left panel of Figure 4a). Placed on filter paper, they looked like "green seeds" compared to fronds (Figure 4b).

### Cloning and sequencing of members of the *Spirodela *APL gene family

**Figure 5:**
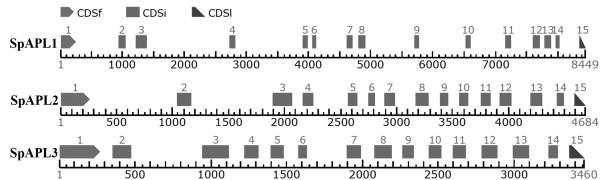
**Structural organization of the large subunit of AGPase (*SpAPL*) genes**. The coding exons were depicted as gray boxes. Introns were depicted as bar scaled by x-axis (bp). CDSf--First (Starting with Start codon), CDSi--internal (internal exon), CDSl--last coding segment (ending with stop codon).

The level of starch accumulation in turions (Figure 2) and the convenience of collecting them from the flask bottom (Figure 4) are key features for biofuel applications as described above. To examine the metabolic regulation of these features, this study seeks to identify key enzymes, whose manipulation at the molecular level could optimize the timing and level of starch production. Common knowledge would then suggest investigating the differential expression of key enzymes in starch biosynthesis. Therefore, we decided to clone the large subunit of the ADP-glucose pyrophosphorylase gene family (*APLs*) from *Spirodela polyrhiza*. Because this gene is very conserved among angiosperms, we used the known *Arabidopsis *protein sequences to design degenerate primers to amplify *APL *coding sequences as described under Methods. Cloned DNA fragments were then sequenced and overlapping fragments were used to reconstruct the entire three cDNA-copies from *Spirodela*. We named them *SpAPL1*, *SpAPL2 *and *SpAPL3 *with Genbank accession numbers of JN180634, JN180635, JN180636. Based on the cDNA sequences primers were then designed to clone the corresponding gene sequences from total genomic DNA as described under Methods. The cloned genes of *SpAPL1*, *SpAPL2 *and *SpAPL3 *were then also sequenced and deposited into GenBank with accessions JN180631, JN180632, JN180633, respectively. After aligning cDNAs with their corresponding genomic sequences, all introns could be identified. Accordingly, all *SpAPLs *consisted of 15 exons and 14 introns (Figure 5). Whereas the coding sequences of the *SpAPL1*, *SpAPL2 *and *SpAPL3 *genes were slightly different in length with 1,554, 1,611, 1,620 bp or 517, 536, and 539 amino acids, respectively, the corresponding genomic regions differed significantly with 8,449, 4,684 and 3,460 bp (Table 1), reflecting intron expansions.

**Table 1:** Gene features of *APL *family

Gene Name	Gene Length (bp)	ORF Length (bp)	Putative Protein Length (aa)	Intron Length (bp)	MITE Length (bp)	Ratio = MITE/Intron
*SpAPL1*	8449	1554	517	6895	2507	0.36
*SpAPL2*	4684	1611	536	3073	659	0.21
*SpAPL3*	3460	1620	539	1840	126	0.07

### Structure and phylogeny of members of the Spirodela APL gene family

The basis for the variation in protein sizes became clear when their primary structures were compared with other known APLs. Sequence alignments of the deduced amino acid sequences of SpAPL1, SpAPL2, and SpAPL3 proteins showed high homology except for their N-terminal regions (Additional file 1: Figure S1). APLs are usually targeted to the plastid through a signal peptide at their amino-terminus. SpAPL2 and SpAPL3 had conserved plastid-targeting signals with cleavage sites at positions 78 and 64 based on TargetP http://www.cbs.dtu.dk/services/TargetP/. The shorter protein of SpAPL1 had a very weak targeting signal and internal deletions similar to the rice APLs. Although there were differences in the amino-terminal regions, the coding sequences from exon 3 to 15 were of the same size and very conserved.

The corresponding introns, however, have diverged significantly in length and composition. Interestingly, comparison to the TIGR Plant Repeat Database [20] indicated that expansion of introns could be largely due to miniature inverted-repeat transposable elements (MITEs). When the MUST system was applied that was used to predict MITEs rather than depending on sequence homology alone, the sequence data suggested then that MITEs had invaded the introns of *SpAPL1*, *SpAPL2*, and *SpAPL3*, comprising now 36%, 21%, and 7% of total intron sequences, respectively (Table 1).

**Figure 6:**
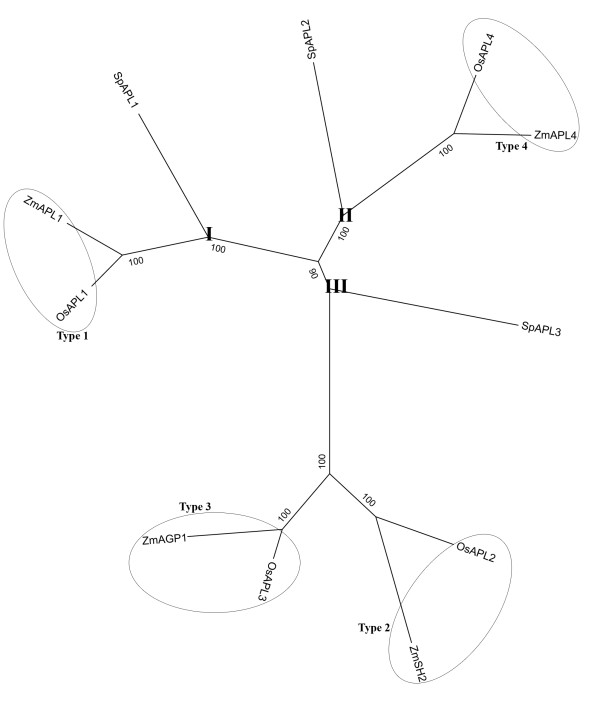
**Phylogenetic tree based on the amino acid sequence alignment of large subunits of AGPase (*APLs*) from *S. polyrhiza *(Sp), *Oryza sativa *(Os), and *Zea mays *(Zm)**. The protein names are those published previously or predicted from CDS: OsAPL1, NP_001051184; ZmAPL1, NP_001106017; SpAPL1, JN180634; OsAPL2, NP_001043654; ZmSH2, NP_001121104; OsAPL3, NP_001056424; ZmAGP1, NP_001105717; SpAPL3, JN180636; OsAPL4, NP_001059276; ZmAPL4, NP_001106058; SpAPL2, JN180635. Three clades were designated APL-I, APL-II, APL-III. The classification of APLs in grasses has previously been published [21].

Using the amino acid sequence alignment of *APL*s from *S. polyrhiza*, rice, and maize, we constructed a maximum likelihood phylogeny of the *APL *family. This phylogenetic tree separated the *APL*s into three main clades: *SpAPL1 *clustered together with the plastidial forms of *OsAPL1 *and *ZmAPL1 *in branch APL-I. *SpAPL2 *shares the branch APL-II with the plastidial forms of *OsAPL4 *and *ZmAPL4*; *SpAPL3 *shares a common ancestor with both plastidial (*OsAPL3 *and *ZmAGP1*) and cytosolic forms (*OsAPL2 *and *ZmSH2*) in rice and maize [21] (Figure 6).

### A structural model of the APLs

**Figure 7:**
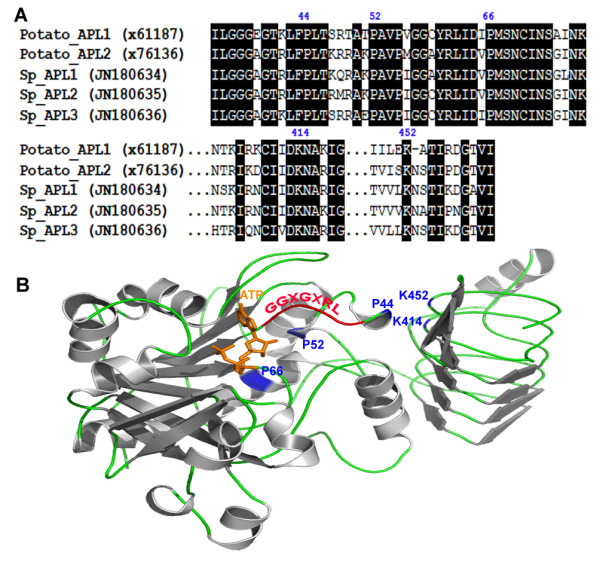
**Amino acid sequence alignment for the regulatory sites of APLs between potato and S. polyrhiza and modeled structure of S. polyrhiza APL1**. **a**) GenBank accession numbers were listed in parentheses. Important proline (P44, P52 and P66) and lysine (K414 and K452) residues critical for allosteric regulation were numbered corresponding to potato AGPase large subunit (x61187). Identical residues were shaded in black. **b**) The N-terminal region containing the putative ATP binding site and the regions containing the putative 3-PGA binding sites of APL1 were modeled by comparison with the known structure of the potato AGPase small subunit (PDB 1yp3) with 54.93% identity. The modeled position of ATP in orange was shown. The α-helix and β-sheet were colored in gray and the loop was in green. Important proline (P) and lysine (K) residues in APL1 were indicated by blue color. The conserved GGXGXRL loop region was in red.

To confirm the inference of their function, three-dimensional structures of SpAPLs were built by using the experimental protein structure (PDB 1yp3) from potato as a suitable template. Amino acid sequence alignment of the regulatory site of APLs from potato and *S. polyrhiza *showed five key conserved residues (P44, P52, P66, K414 and K452) (Figure 7a) in all three SpAPLs. Molecular modeling analysis of APLs suggested a critical role of APLs for allosteric regulation in this region with binding sites for ATP and 3-PGA (Figure 7b). P44 was important for accommodating ATP phosphate groups, as it was located between a conserved GGXGXRL loop region and the strongly conserved "PAV" region, which involved catalysis and allosteric regulation [22]. P52 was predicted to be located in flexible loops close to the lysine residues (K414 and K452), while P66 lied in a helix. Site-directed mutagenesis of the P52 and P66 in potato showed dramatic changes in affecting enzyme regulatory properties, while P44 mutants resulted in a nearly catalytically inactive enzyme [23]. K414 and K452 were shown to be involved in the increase of the affinity for the activator 3-PGA [22,24]. Model structures of APL1, APL2 and APL3 were identical in these features. Therefore, only APL1 was shown in Figure 7 as an example.

### Expression patterns of APL genes in developing turions

With three different gene copies present in *Spirodela polyrhiza*, the question arises how each enzyme is expressed temporally during turion formation. We therefore isolated total mRNA from leaf-like tissue 0, 1, 2, 3, 5, and 7 days after the addition of ABA. To measure expression of each APL gene copy, we applied qPCR to mRNA samples using specific primer pairs to distinguish between transcripts from each gene. Expression of *SpAPL2 *and *SpAPL3 *dramatically increased two-and 10-fold, respectively, as turion development was initiated (1-3 days). Furthermore, there seemed to be a difference in the expression of *SpAPL2 *and *SpAPL3*. *SpAPL2 *was critically in the first phase of induction, whereas *SpAPL3 *seemed to replace *SpAPL2 *in a second burst of activity. There was no obvious increased expression of *SpAPL1 *after ABA induction. Indeed, *SpAPL1 *appeared to be more active in initial fronds compared to *SpAPL3 *(0 days of ABA application). When turions went into mature phase (after 5 days), the expression of all *SpAPLs *was leveling off (Figure 2b).

## Discussion

We began dissecting the process of turion formation in duckweeds. Usually turion development occurs in late summer or early autumn because of starvation and lower temperatures [25]. *Spirodela *turions can also be induced under controlled laboratory conditions by increasing the concentration of ABA in the growth medium [11,13], decreasing temperature [15], or depriving phosphorus in the medium [12]. Here, we have taken advantage of ABA as an inducer and could reproduce the morphological changes that occur during turion formation. Turions are germinated into new fronds in the presence of light and nitrogen in the following spring using starch storage as an energy source [26,27]. Therefore, the drastic starch accumulation during turion formation marks a turning point in the switch process from low-starch fronds to high-starch turions.

The reported contents of starch varied from 14% to 43% depending on the species, developmental states (fronds, resting fronds, or turions) [28] and tested methods [5,29]. Starch content could even go up to 75% of the dry weight in resting fronds of *Spirodela oligorrhiza *(renamed into *Landoltia punctata*) growing in phosphor-deficient cultures [12], a level that is comparable to cereal seeds of corn, sorghum and wheat [30]. Even though regular fronds have as low as 16% starch in dry mass, turions of *S. polyrhiza *can reach up to 62% starch [25]. Our use of exogenous ABA produces the same developmental switch, as the different morphological features are easily distinguishable. The switch is rapid, providing advantages for biochemical and physiological analysis [13]. We obtained 60.1% starch from dry mass after 2 weeks of ABA induction (Figure 2a), which is comparable to the Henssen's study. The size of mature starch grains from turions was around 4 μm in diameter as estimated by TEM (Figure 3e and 3f), whereas starch grains from wheat, corn and rice reach a size of 30 μm, 25 μm and 20 μm, respectively [31]. In a different study, the size of starch granules illuminated by red light for different times have also been measured using SEM scans arriving at similar values [32]. Interestingly, it has been suggested that smaller starch granules are more easily hydrolyzed into sugars than larger ones, regardless of botanical source [33]. After 72 h of continuous irradiation, the sizes of starch granules in turions are significantly reduced to about 1.5 μm [32]. Although duckweeds might have adapted to rapidly switch back to a growth phase faster than seed plants, this property also might provide a more efficient way for producing bio-ethanol than from maize.

Amyloplasts in non-photosynthetic tissue, such as seeds, roots, and stems, which lack chlorophyll and internal membranes, are the main organelles responsible for the synthesis and storage of starch granules in most plants. However, turions remain green or dark-green throughout their development (Figure 1b and 4b). The plastids in turions, where starch synthesis takes place, still retain abundant stacks of thylakoids (Figure 3e and 3f). It therefore suggests that chloroplasts with a simple structure as in duckweeds can function both as source and sink. The starch-storing plastids of turions are directly derived from chloroplasts, and retain chloroplast-like characteristics throughout their development. This adaptation greatly saves energy by directly depositing sucrose generated from photosynthesis into starch storage without the need for transport through a vascular system and the use of a glucose phosphate transporter [21]. A similar system exists also in a non-aquatic plants such as pea embryos, where starch-storing plastids also directly originate from chloroplast [34,35]. Moreover, using TEM light-induced degradation of starch granules in turions of *Spirodela polyrhiza *also exhibited a transition from amyloplasts to chloroplasts [32]. Both studies would demonstrate that differentiation from chloroplast to amyloplast could be reversed based on physiological changes. Indeed, the cell structure of turions appears to be well organized for its function. Its lack of intercellular air space and presence of smaller vacuoles allow them to survive in deep water, where the temperature is more moderate than on the surface. The numerous starch grains provide a bank of energy when turions germinate in the following spring. This life cycle is also consistent with starch content in fronds and turions.

Because starch biosynthesis is an important feature for the developmental switch from fronds to turions, it also provides us with the first entry point to dissect the developmental regulation of turion formation. Therefore, we reasoned that the first step in this line of investigation consists of the identification and characterization of key regulatory genes known in starch biosynthesis, which are the ADP-glucose pyrophosphorylases. We successfully cloned three copies of *APLs *of *Spirodela polyrhiza*. *APLs *are expressed in different organs of grass species, type 1 in leaves, type 2 and type 3 in seeds, and type 4 in both seeds and leaves [21,36]. Based on phylogeny and spatial expression of *SpAPLs *(Figure 6 and 2b), they have their homologs in grass species. *SpAPL2 *and *SpAPL3 *are active in turions, while *SpAPL1 *is expressed at a higher level in fronds. The transcript level of *SpAPL2 *and *SpAPL3 *are active at an early phase of turion formation, while all transcript level of *SpAPLs *decline towards the end phase. It could account for the inhibition of total RNA synthesis after 3 days in ABA, which leads to the shutdown of all primary processes and onset of the dormant state [37]. Analysis of networks of gene expression during *Arabidopsis *seed filling has also shown that expression of carbohydrates occurred early in seed development [38]. Noticeably, the transcription of *SpAPL1 *and *SpAPL2 *is suppressed right after one day of ABA addition, which is quite consistent with previous findings that ABA could inhibit DNA, protein, and RNA synthesis during turion development [37]. But this inhibitory effect of ABA during turion development is selective for that the synthesis of certain turion specific proteins increases [37]. Indeed, the pattern of expression was consistent with a rate-limiting role for this protein in starch biosynthesis. Furthermore, the regulation of gene copies underwent divergence and probably sub-functionalization to permit metabolic differentiation.

In plants, ADP-glucose pyrophosphorylases consist of large and small subunits that share many amino acids due to the proposed origination from a common ancestral gene [39]. For example, APLs and APSs, which make up the heterotetrametic potato enzyme, share significant sequence homology (53% identity and 73% similarity) [40]. Here we selected the large subunit for our analysis because we made the assumption that both are coordinately expressed and that the large subunit should suffice as a marker of the developmental switch between frond and turion stage of the life cycle. Furthermore, the current sequencing of the entire genome will provide an opportunity to locate the gene copies of the small subunit as well. The model structure of the large subunit confirms that N-and C-terminal regions of the SpAPLs are essential for the allosteric regulatory properties of the heterotetrameric enzyme AGPase (Figure 7b) [23]. Even though APLs are considered as a catalytic-disabled subunit, the ability of binding effectors (3-PGA) and substrates (ATP) is likely to undergo a conformational transition similar to the APSs during its catalytic cycle [41].

Phylogenetic analysis showed that *SpAPL1 *and *SpAPL2 *descended from common ancestors of the plastidial form Type 1 and Type 4 of the grasses, respectively, while *SpAPL3 *shares the same branch with the ancestor of cytosolic Type 2 and plastidial Type 3 of grasses (Figure 6) [17]. Studies suggest that cytosolic Type 2 in grass evolved from a duplication of an ancestral gene encoding a Type 3 plastidial *APL *by loss-of-function of the transit peptide cleavage site [21]. A similar process might have taken place in *Spirodela*, where *SpAPL1 *does not exhibit a clear transit peptide. Interestingly, the opposite seems to be true for *SpAPL3*, which clusters with cytosolic Type 2 *APL*s, but encodes a transit peptide. Based on this, we classify it as a plastidial Type 3 *APL *of the grasses. The phylogenetic relationship will become clearer when we know whether these copies are clustered or dispersed in the *Spirodela *genome. Interestingly, there is differential invasion of MITES in the introns of these genes with the most pronounced invasion in the *SpAPL1 *gene (Table 1). This is reminiscent of the grasses, where one of the smallest genomes, rice, had a relative high percentage of MITEs (13.3% of all repeat elements compared to 0.4% in maize), but low retrotransposon content (59.5% compare to 92.7% in maize). *Spirodela polyrhiza *was namely chosen for sequencing because of its small genome size. Given the genome size variation among *Lemnoideae*, perhaps a similar relationship of genome size and MITEs exists among *Lemnoideae *as has been found in grass species [42].

## Conclusion

In summary, turions of *S. polyrhiza *contain high starch content, small size of starch granules, and low lignin proportion, which provides a solid foundation for developing them as an alternative biofuel source. For further investigation of the role of *SpAPL2 *and *SpAPL3 *genes in starch synthesis, studies using transgenic plants will be needed.

## Methods

### Plant material and growth conditions

For our studies we chose *S. polyrhiza *(Sp) 7498 because this will serve as a reference genome for the *Lemnoideae*. One cluster of 3-5 fronds was aseptically transplanted into half-strength Schenk and Hildebrandt basal salt mixture (Sigma, S6765) with 1% sucrose liquid medium at pH 5.8. The cultures were kept in chamber maintained at 100 μmol.m^-2^.s^-1 ^and 23°C through a 16 h-light, 8 h-dark photoperiod. After a couple of days' growth, 1 μM abscisic acid (ABA, Sigma, A1049) was added.

### Microscopic analysis of frond and turion

Vegetative fronds without ABA treatment and turions with 14 days ABA treatment were fixed, embedded, and dehydrated as described [43]. Samples were fixed in 5% glutaraldehyde in 0.1 M sodium cacodylate buffer, pH 7.4, containing 2% Suc in a 2-ml tube at 4°C overnight and another 3 h at room temperature. Rinsed by 0.1 M sodium cacodylate buffer, they were postfixed in buffered 1% osmium tetroxide at 4°C overnight followed by dehydration in a graded series of acetone washings. The dehydrated samples were then embedded in epon resin. The 1 mm-thick sections were picked up on a glass slide, stained with methylene blue and scoped with a light microscope. For transmission electron microscopy (TEM), 90 nm-thin sections were cut on a Leica EM UC6 ultramicrotome, stained with a saturated solution of uranyl acetate and lead citrate and scoped at 80 kV with a Philips CM 12 transmission electron microscope.

### Determination of starch content of developing turions

One hundred milligrams of fresh sample tissues were taken from a time course of 0 (no ABA), 1, 2, 3, 5, 7, 10, 14 days of ABA treatment and flash frozen in liquid nitrogen. Before 7 days, the whole plants including both mother and daughter fronds were collected. After 7 days, the developed turions were separated from mother fronds and collected, when they sunk to the bottom of flask (Table 2). Three biological replicates were done for each time point. The quantification of starch content was determined colorimetrically following manufacturer's protocols of a "total starch assay" procedure (amyloglucosidase/α-amylase method) (Megazyme, K-TSTA). We used water as a blank control and D-glucose as a standard. Dry weight was counted by 500 mg fresh tissue after incubation in 65°C chamber for 24 h.

**Table 2:** Sample collection for starch analysis and *APLs *expression quantification

Days in ABA	Samples for Testing Starch Content	Size of fronds or turions (mm) for Analysis *APLs *Expression	Characterization of Developing Turions
0	Whole plants	~ 0.5-0.7	Light green
1	Whole plants	~ 1	Light green
2	Whole plants	~ 1.5	Light green
3	Whole plants	~ 2	Dark green
5	Whole plants	~ 2	Dark green
7	Only turions	~ 2	Dark green, sink at the bottom
10	Only turions	no collection	Dark green, sink at the bottom
14	Only turions	no collection	Dark green, sink at the bottom

### Genomic DNA and total RNA isolation

Total genomic DNA was extracted from whole plant tissue by the CTAB method [44]. Considering that only daughter fronds shorter than 0.7 mm in length respond to ABA treatment and undergo turion formation after ABA treatment [16], developing turions only with specific sizes were collected at their developmental stages after 0 (no ABA addition), 1, 2, 3, 5, 7 days of ABA treatment, respectively, for quantification of *APL *gene expression (Table 2). For each time point we used again three biological replicates. High-quality total RNA was extracted with RNeasy Plant Mini Kit (Qiagen, 74904). The on-column DNase I was used to remove contaminating genomic DNA (Qiagen, 79254). The RNA quality and quantity were confirmed by analysis with Nanodrop 1000 (Nanodrop Technologies, Wilmington, DE). First-strand cDNA synthesis of all samples was generated by kit of SuperScript™ III First-Strand Synthesis System for RT-PCR (Invitrogen, 18080) using oligo-dT as primer.

### Retrieval of APL genes and CDS sequence

The conserved domains of APL proteins of *Arabidopsis *were used to set up degenerate primers. Degenerate PCR reactions were done with templates of cDNA extracted from samples of 3 days of ABA treatment. The program was: 35 cycles of 94°C 30 s, 50°C 30s and 72°C 1 min. PCR products were cloned into the pGEM-T Easy Vector (Promega) and DNA fragment sequences were determined using the ABI 3730XL platform. Gene specific primers were designed based on the sequence of the cloned DNA to perform 5' and 3' RACE using the SMARTer™ RACE cDNA Amplification Kit (Clontech, 634923). The RACE-ready cDNA was also generated from total RNA of samples treated 3 days with ABA. RACE reactions were performed under the following program: 5 cycles of 94°C 30 s and 72°C 2 min; 5 cycles of 94°C 30 s, 70°C 30s and 72°C 2 min; 25 cycles of 94°C 30 s, 68°C 30 s and 72°C 2 min. The RACE products were also cloned and sequenced. The full-length cDNA was confirmed with primers designed from 5' end of the 5' RACE sequence and the 3' end of the 3' RACE sequence. The same primers were used to amplify corresponding gene sequences using genomic DNA as template. Because of the size of the genes we used Expand Long Range dNTPack (Roche, #04829042001). The thermal cycling conditions were: 10 cycles of 94°C 15 s, 55°C 30 s and 68°C 9 min; 25 cycles of 94°C 15 s, 55°C 30 s and 68°C 9 min with 10 more seconds for each cycle. Initially, primer sequences derived from APL cDNA were used to sequence genomic DNA. Subsequently, primers derived from genomic sequences were used in iterative rounds of sequencing until sufficient coverage was achieved. The sequences were assembled and analyzed with DNASTAR. MUST system, which tested the existence of a pair of terminal inverted repeats (TIRs) and a pair of direct repeats (DRs) [45] was used to predict miniature inverted-repeat transposable elements (MITEs) in *APL *introns. All successful primers were listed in Additional file 2: Table S1.

### Phylogenetic studies

An unrooted maximum likelihood phylogenetic tree was determined by using the MEGA 5 program [46] based on the amino acid sequence alignments under the WAG model with 1000 bootstrap replications. The corresponding subunit sequences from rice and maize were downloaded from GenBank.

### Modeling of the three-dimensional structures

Sequences of the APL regulatory sites from potato and *S. polyrhiza *were aligned using MEGA 5. Homology modeling studies were performed using the Swiss Model server (http://swissmodel.expasy.org/) [47] and structures were visualized and prepared by an open source program PyMOL (The PyMOL Molecular Graphics System, Version 0_99rc6, Schrödinger, LLC.). The sequence used was that of SpAPL1, SpAPL2 and SpAPL3. The chosen suitable template was homodimeric AGPase of potato (PDB 1yp3) [22] for which X-ray structure information was available, showing more than 52% sequence homology with SpAPLs. Key proline (P44, P52 and P66) and lysine (K414 and K452) residues were numbered based on AGPase large subunit of potato (x61187) [23]. Only APL1 modeled structure was shown in Figure 7b as representative for the sake of simplicity.

### Expression analysis of APL genes

Alignment of full length of cDNAs produced unique regions at the 5' UTR to design primers for qPCR (Additional file 2: Table S1). qPCR was performed for 0, 1, 2, 3, 5, 7 day of ABA treatment. All cDNAs were made with 2 μg of RNA using the SuperScript^® ^III First-Strand Synthesis System kit (Invitrogen, 18080-051). cDNAs were diluted 20-fold and Real-time PCR was performed by using the iQTM SYBR Green Supermix (Biorad, 170-8880) following the manufacturer's standard instructions. All qPCRs were performed in triplicates. The relative quantification of each gene expressional level was calculated by calibrating CT values normalized to a standard dilution series over all samples assayed [48].

## Authors' contributions

WW designed experiment, analyzed data and wrote the manuscript. JM supervised the work, interpreted data with WW, and revised all versions of the manuscript. All authors read and approved the final manuscript.

## Supplementary Material

Additional file 1 **Figure S1**. Multiple alignments of the deduced amino acid sequences of APL proteins from S. polyrhiza (Sp) and Oryza sativa (Os). Dashed lines indicate gaps introduced to maximize alignment. Click here for file

Additional file 2 **Table S1**. Primers for cloning, sequencing and quantifying APLs expressions. *Primers were cited from [49]. Click here for file
