# Supplementary figures and images for: Analysis of ADP-glucose pyrophosphorylase expression during turion formation induced by abscisic acid in Spirodela polyrhiza (greater duckweed)
Source: BMC Plant Biol. 2012 Jan 11;12:5. doi: 10.1186/1471-2229-12-5 (PMC3268088; doi:10.1186/1471-2229-12-5)

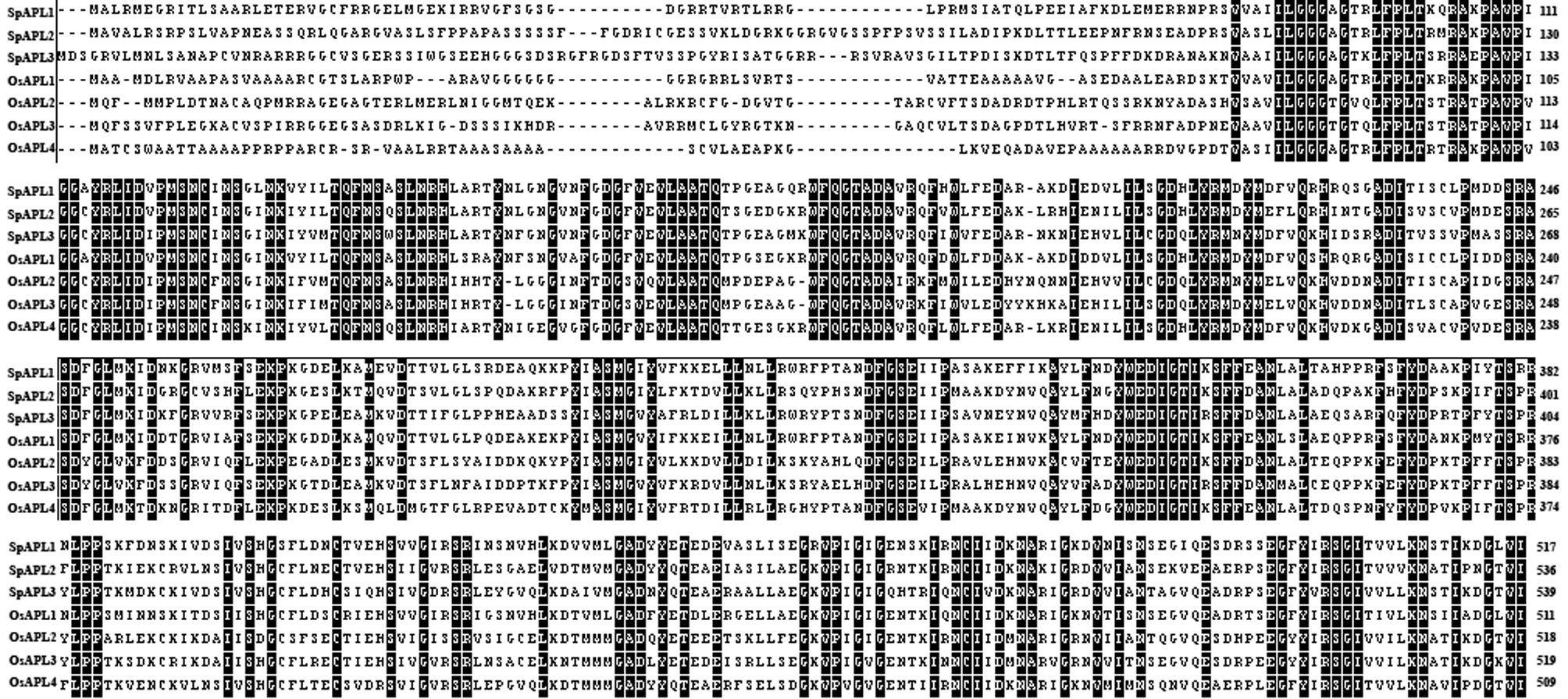

Supplement: Additional file 1 — Figure S1. Multiple alignments of the deduced amino acid sequences of APL proteins from S. polyrhiza (Sp) and Oryza sativa (Os). Dashed lines indicate gaps introduced to maximize alignment. [file 1471-2229-12-5-S1.JPEG]
